# RoadTopo-SAM:Topology-Aware Vectorized Road Network Extraction from High-Resolution Satellite Imagery of Open-Pit Mines

**DOI:** 10.3390/s26206459

**Published:** 2026-10-12

**Authors:** Bo Zou, Yi Jia, Ruifei Zhu, Jie Wen, Shixu Song, Yilong Meng, Nan Lu

**Affiliations:** Chang Guang Satellite Technology Co., Ltd., Changchun 130102, China; celiangzou@163.com (B.Z.); zhuruifei1105@163.com (R.Z.); wenjie1164@jl1.cn (J.W.); jlssx1992@126.com (S.S.); myl970820@163.com (Y.M.); lunan@jl1.cn (N.L.)

**Keywords:** open-pit mine, road extraction, vectorized road network, topology reasoning, Segment Anything Model, high-resolution remote sensing

## Abstract

Open-pit mine road extraction is of significant value for intelligent navigation, yet it faces significant challenges due to unstructured terrain, spectral homogeneity, and physical occlusions. Traditional semantic segmentation methods frequently result in fragmented road masks. This paper proposes RoadTopo-SAM, a topology-aware vectorized mine road extraction framework based on the Segment Anything Model (SAM), transforming road extraction from pixel-level classification to a graph generation task. The framework includes a geometry decoder guided by a topology-constrained loss to generate graph nodes, and a Transformer-based topology reasoning module to explicitly infer long-range connections between nodes. Additionally, a phased differential fine-tuning strategy is employed to prevent the degradation of pre-trained generic representations while effectively adapting the visual foundation model to mining scenarios. Experiments on 0.5-m resolution “Jilin-1” imagery of Tibetan copper mines and the Nanfen iron mine demonstrate that the proposed method achieves state-of-the-art performance with a TOPO F1 of 90.48% and an APLS of 45.35%. Significance testing indicates its superiority over traditional baselines. Cross-domain region-level inference further validates the model’s robustness in macroscopic topological connectivity, providing an effective solution for vectorized mine road mapping.

## 1. Introduction

Alongside advanced object detection and scene classification [1,2,3], road network extraction serves as a fundamental task in high-resolution remote sensing imagery interpretation, playing a pivotal role in geospatial data updating, intelligent transportation navigation, and mineral resource security monitoring [4,5,6]. The routine Earth observation capabilities achieved by Chinese sub-meter optical remote sensing satellites, such as the Jilin-1 (JL-1) constellation and the Gaofen (GF) series, have substantially propelled the advancement of automated, fine-grained road interpretation [7,8]. Nevertheless, existing road extraction benchmarks and deep learning models are predominantly tailored for urban road networks and relatively structured suburban highway systems [9,10]. When directly deploying these algorithms to open-pit mining characterized by high altitudes, severe topographic relief, and complex excavation geometries—such as porphyry copper mines in the Tibetan Plateau and deep-concave stepped open-pit iron mines—their segmentation accuracy and topological continuity deteriorate substantially [11,12,13]. In these mining environments, unpaved haul roads typically wind along mountainous terrain or open-pit mining benches, exhibiting narrow geometric widths of merely a few pixels and highly tortuous trajectories [14,15]. Furthermore, these haul roads share highly homogeneous spectral characteristics with adjacent overburden dumps, safety berms, and tailings. Airborne dust generated by heavy-duty mining trucks further degrades the radiometric contrast between the road corridors and the background terrain. Consequently, conventional semantic segmentation networks frequently suffer from topological disconnections and false-positive misclassifications around sharp curves, intersections, and shadow-occluded regions [16,17].

To mitigate such adverse imaging conditions, the recent literature offers advanced image restoration and enhancement techniques. Physically motivated priors, such as the inverted haze density correction prior (IHDCP) [18], are highly effective for single-image dehazing. Temporal redundancy can be exploited via deep video dehazing with semantic segmentation [19]. Furthermore, nighttime and low-light degradations have been addressed using Bayesian-based fractional-order variational models [20] and hybrid-regularization schemes like VNDHR [21]. Additionally, to overcome inherent spatial and spectral resolution limits in complex scenes, recent advances in image preprocessing, such as unsupervised super-resolution [22,23], hyperspectral unmixing [24], and robust data compression [25], offer complementary perspectives for enhancing foundational image quality prior to feature extraction.

In the evolution of road extraction methodologies, early studies predominantly relied on local edge detection, mathematical morphology, and active contour models [26,27]. These manually designed rule-based approaches are sensitive to illumination variations and background clutter, exhibiting limited cross-scene generalization capability. The introduction of convolutional neural networks (CNNs) substantially enhanced feature representation capabilities. Representative encoder-decoder architectures, such as U-Net [28], D-LinkNet [29], and SegNet [30], improved the topological connectivity of long-range road networks by leveraging dilated convolutions and multi-scale feature aggregation [31,32]. However, the localized receptive fields and weight-sharing mechanisms of standard convolutions inherently constrain their capacity to explicitly model long-range spatial dependencies, i.e., the structural relationships and connectivity between distant road segments belonging to the same network. When mine haul roads are occluded, the network relies solely on locally visible texture cues for inference, hindering the complete reconstruction of the underlying road topology [33,34]. Furthermore, successive downsampling operations severely aggravate the loss of fine-grained spatial details along narrow road branches. Consequently, unpaved haul roads measuring only a few pixels in width struggle to preserve discriminative localization cues in deep feature maps after hierarchical pooling [35,36].

Vision Transformers (ViTs) and emerging state-space models (e.g., Mamba) overcome the limited receptive fields of CNNs via global contextual mechanisms and have been widely adopted in remote sensing tasks [37,38,39,40]. The Segment Anything Model (SAM) introduced by Kirillov further demonstrates impressive direct transfer generalization capabilities based on large-scale visual pre-training. However, adapting these visual foundation models directly to mine haul road extraction faces two critical bottlenecks. First, mainstream adaptations formulate road extraction strictly as a dense pixel-level semantic segmentation task. This paradigm fundamentally neglects the inherent topological graph structure of road networks [41]. Consequently, even with powerful semantic features, pure segmentation models struggle to guarantee the physical connectivity and navigational validity of the extracted roads, frequently generating fragmented masks under severe dust occlusion [42,43]. Second, directly applying full fine-tuning to parameter-heavy foundation models tends to degrade the generic representations learned during pre-training, a phenomenon known as catastrophic forgetting, and to cause overfitting under data-scarce mining regimes [44].

Beyond the architectural paradigms, evaluating and optimizing road extraction models in open-pit mines suffer from substantial domain gaps and metric inadequacies [45]. When transferring a pre-trained model to complex open-pit mine characterized by deep-concave multi-tiered bench slopes and reddish-brown backgrounds, the texture distribution shifts drastically. Conventional transfer learning strategies often lead to suboptimal convergence, necessitating a delicate balance between retaining generic visual priors and adapting to specialized geomorphological features. Furthermore, traditional pixel-level evaluation metrics are highly sensitive to road width variations and fail to severely penalize topological disconnections [46]. A model predicting wide but disconnected road segments might obtain a high IoU, yet such outputs are entirely invalid for actual mining truck routing and navigation [47]. Therefore, shifting the paradigm from pixel-wise mask prediction to vectorized graph topology generation, coupled with domain-adaptive fine-tuning and graph-level connectivity evaluation, has become an urgent imperative for advancing mine road extraction.

We propose a topology-aware vectorized road network extraction framework specifically designed for unstructured open-pit mines. By reformulating road extraction from a pixel-wise semantic segmentation task into a graph generation paradigm, our approach effectively mitigates boundary degradation, topological disconnections, and domain shifts. The principal contributions of this work are summarized as follows:

- We design a geometry decoding branch constrained by a joint topological loss (clDice). This preserves the skeleton connectivity of narrow, dust-occluded mining roads and generates sparse candidate graph vertices, alleviating the fragmentation issues inherent in conventional segmentation.
- To handle the pseudo-connections caused by intricate mountainous terrain, we propose RoadTopo-SAM, a topology reasoning module. By aggregating semantic features and relative coordinate offsets, the multi-head self-attention mechanism captures multi-hop spatial dependencies, facilitating the prediction of valid physical edges between candidate vertices.
- We introduce a phased differential unfreezing strategy for the visual foundation model. This strategy mitigates catastrophic forgetting while adapting deep semantic representations to the unstructured geological domains of open-pit mines, reducing the instability often associated with adversarial domain adaptation.
- Moving beyond traditional area-based metrics which exhibit bias towards variable road widths, we employ an evaluation system incorporating TOPO F1 and Average Path Length Similarity (APLS). Supported by Wilcoxon signed-rank testing, we provide a statistically grounded benchmark for unpaved road navigation validity.

## 2. Method

### 2.1. Overall Architecture

**Figure 1:**
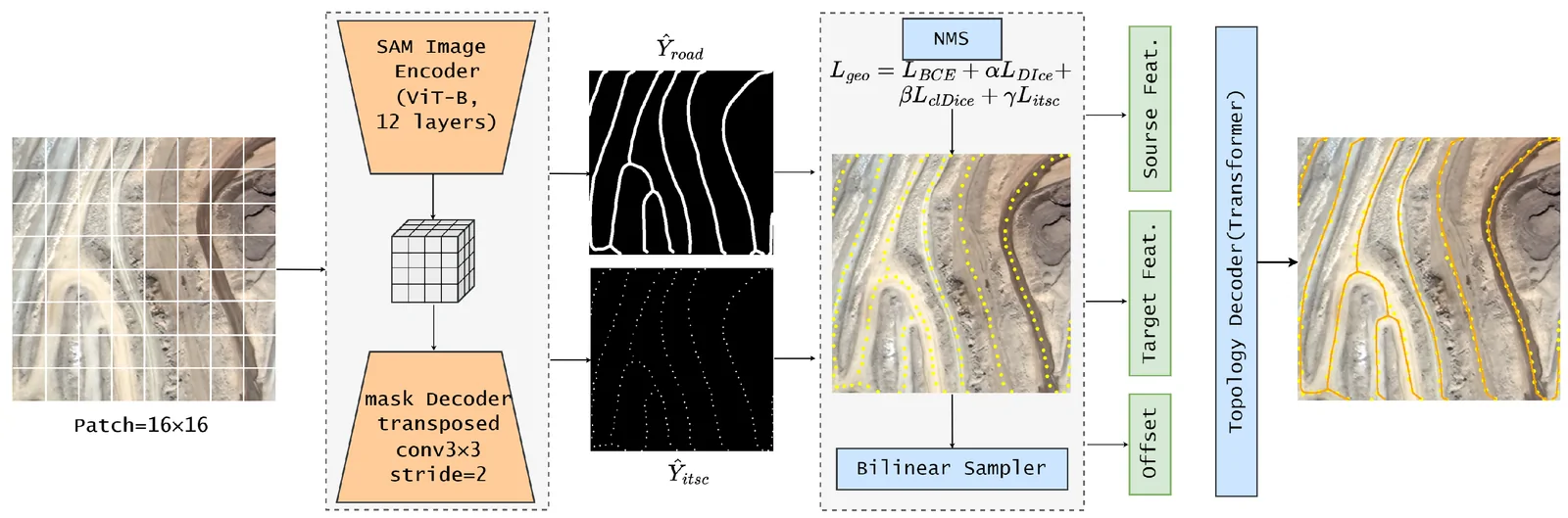
Overall architecture of the proposed RoadTopo-SAM.

Open-pit mine roads typically exhibit highly unstructured characteristics, including blurred boundaries and severe occlusion by mining equipment or airborne dust. Consequently, conventional pixel-level semantic segmentation methods, even when supplemented with heuristic post-processing, struggle to preserve the topological connectivity of road networks. Motivated by the generalizable visual representations of the Segment Anything Model (SAM) image encoder, this study introduces RoadTopo-SAM, a vectorized road graph extraction framework tailored for unstructured mining environments, explicitly modeling the road network as a graph G=(V,E). Given a high-resolution remote sensing image of a open-pit mine, denoted as $I\in R^{H\times W\times 3}$, where *H* and *W* represent the spatial height and width of the image respectively, the model initially extracts deep semantic features via a fine-tuned visual foundation model. The network architecture is subsequently decoupled into two core branches: topology-aware geometry extraction and Transformer-based topology reasoning, as shown in Figure 1.

### 2.2. Visual Foundation Model-Based Feature Encoding

**Figure 2:**
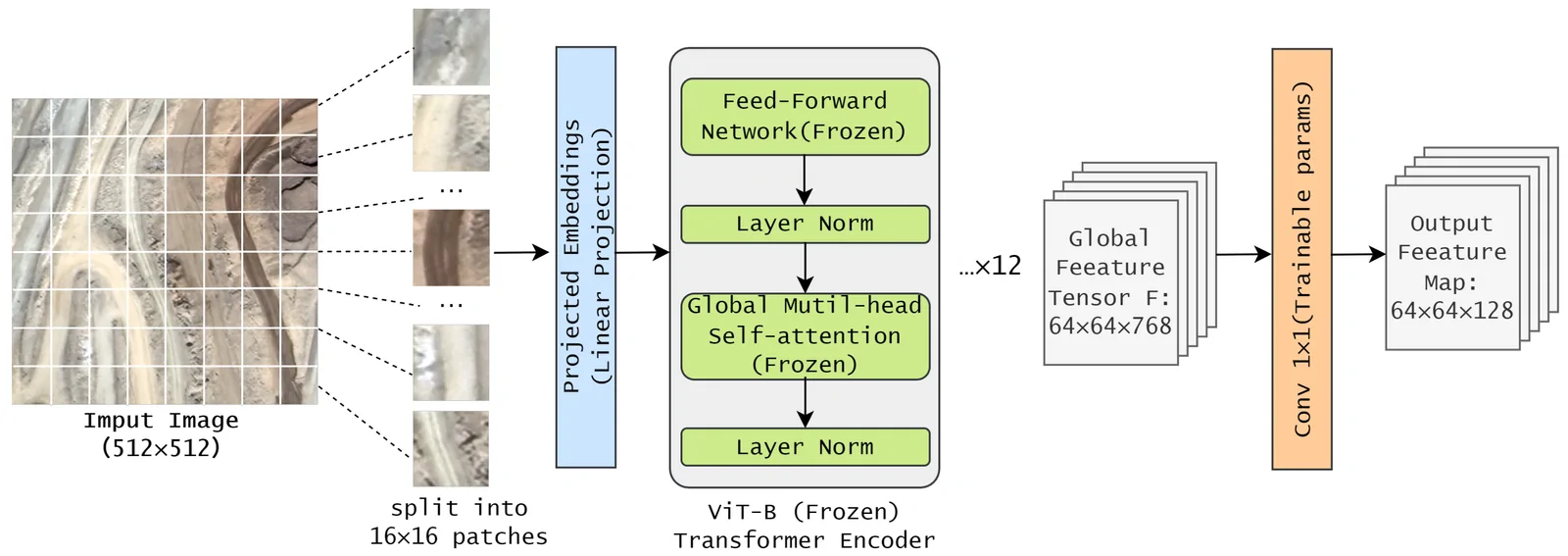
Feature encoding architecture leveraging pre-trained visual foundation models.

Mine roads exhibit high spectral and textural similarities with adjacent bare soil and overburden dumps. Restricted by localized receptive fields, conventional convolutional neural networks struggle to extract continuous semantic features in these challenging regions. Consequently, the image encoder of the Segment Anything Model (SAM) is introduced as the visual foundation feature extractor in this study. This image encoder is built upon the standard Vision Transformer (ViT) architecture, as shown in Figure 2. The input imagery is initially resized to a fixed resolution of 512×512 and subsequently partitioned into non-overlapping patches of 16×16 pixels. These patches are projected into one-dimensional embedding vectors and fed into a 12-layer Transformer network equipped with global multi-head self-attention, omitting the windowed attention mechanism. Following the forward propagation through the encoder, the input image is transformed into a global feature tensor *F*:

(1) $$F=\text{SAM}\_\text{Encoder}\left(I\right),\;F\in R^{32\times 32}\times D_{\text{feat}}$$

Given the fixed input resolution of 512×512, the spatial dimensions of the resulting tensor are 32×32. The variable $D_{\text{feat}}$ denotes the feature dimension. In practice, the original output dimension of 768 from the SAM ViT-B backbone is reduced to 128 via a 1×1 convolution. This robust feature representation demonstrates a strong capacity to bridge regions obscured by airborne dust and shadows, providing essential structural priors for subsequent road geometry extraction and topology reasoning.

### 2.3. Topology-Constrained Geometry Mask Decoding and Vertex Generation

#### 2.3.1. Geometry Mask Prediction

The geometry decoder consists of four stacked transposed convolution layers with a stride of 2. This structure progressively restores the spatial resolution of the feature maps to the original input dimensions, ultimately yielding a two-channel probability prediction map:

(2) $$\left({\hat{Y}}_{\text{road}},{\hat{Y}}_{\text{itsc}}\right)={\text{Decoder}}_{\text{geo}}\left(F\right)$$

where ${\hat{Y}}_{\text{road}}\in {\left[0,1\right]}^{H\times W}$ represents the road probability, and ${\hat{Y}}_{\text{itsc}}\in {\left[0,1\right]}^{H\times W}$ indicates the probability of road intersections or bifurcations.

#### 2.3.2. Joint Topological Constraint Loss

**Figure 3:**
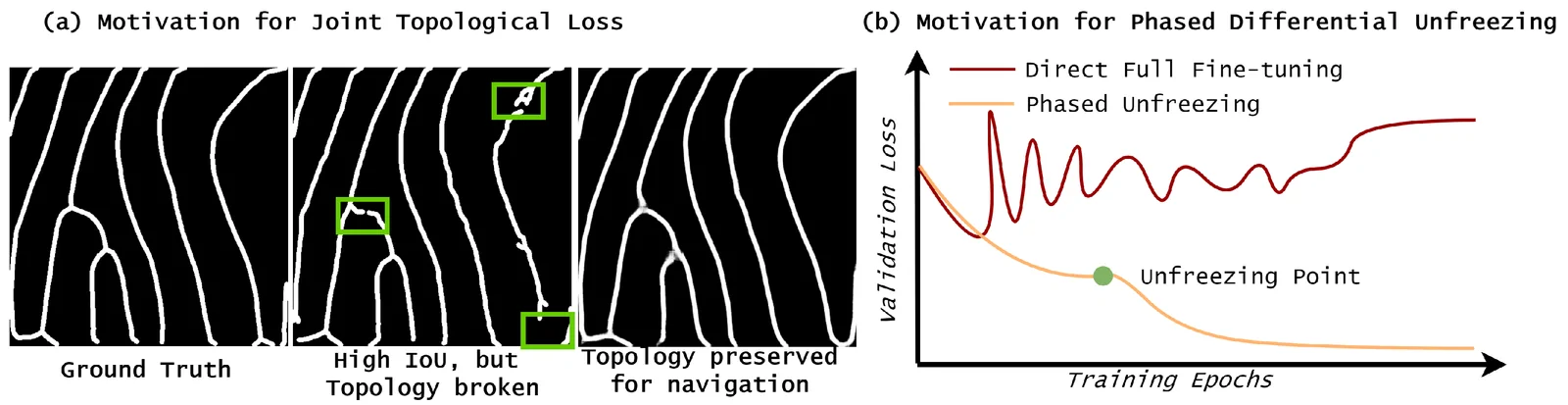
Visualizations of the framework’s optimization motivations. (**a**) Comparison illustrating the necessity of the joint topological loss to prevent critical route disconnections. (**b**) The necessity of the phased differential unfreezing strategy to mitigate catastrophic forgetting during foundation model adaptation.

A purely region-based objective is weakly informative for mine roads that are often only a few pixels wide. As illustrated in Figure 3a, a prediction that covers the road area but breaks its centerline incurs only a modest penalty under standard pixel-wise losses, although the resulting fragmented network is functionally unusable for routing. Therefore, a term that explicitly supervises the road skeleton is required.

To address this, the geometry branch is optimized with a joint loss that combines region overlap with a centerline Dice term. The total loss for the geometry extraction stage is defined as:

(3) $$L_{\text{geo}}=L_{BCE}\left({\hat{Y}}_{\text{road}},Y_{\text{road}}\right)+\alpha L_{\text{Dice}}\left({\hat{Y}}_{\text{road}},Y_{\text{road}}\right)+\beta L_{\text{clDice}}\left({\hat{Y}}_{\text{road}},Y_{\text{road}}\right)+\gamma L_{\text{itsc}}$$

where $L_{\text{itsc}}=L_{BCE}\left({\hat{Y}}_{\text{itsc}},Y_{\text{itsc}}\right)$. The ground truth $Y_{\text{itsc}}$ is automatically generated from the centerline bifurcations of the road labels. Specifically, this generation follows a three-step procedure: (1) the binary road mask $Y_{\text{road}}$ is skeletonized by morphological thinning until a single-pixel-wide centerline remains; (2) every centerline pixel is examined within its eight-connected neighborhood, and is recorded as a branch or intersection point if the circular traversal of that neighborhood contains more than two transitions between road and background; (3) the resulting point set is dilated and then refined by non-maximum suppression (NMS). This ensures that every true bifurcation contributes exactly one compact labeled region consistent with the resolution of the decoder output. The $L_{\text{clDice}}$ term is specifically formulated as:

(4) $$L_{\text{clDice}}\left({\hat{Y}}_{\text{road}},Y_{\text{road}}\right)=\frac{2\sum S_{\hat{Y}}\cdot S_{Y}+\epsilon }{\sum S_{\hat{Y}}+\sum S_{Y}+\epsilon }$$

$S_{\hat{Y}}$ and $S_{Y}$ denote the soft skeletons of the predicted probability map and the ground truth label, respectively, which are obtained through soft erosion and soft dilation operations. During the early stages of training, a linear warm-up strategy is applied to the weights α and β:

(5) $$\alpha \left(t\right)=\alpha_{\text{max}}\cdot \text{min}\left(1,\frac{t}{T_{\text{warm}}}\right),\;\beta \left(t\right)=\beta_{\text{max}}\cdot \text{min}\left(1,\frac{t}{T_{\text{warm}}}\right)$$

where $T_{\text{warm}}$ denotes the number of warm-up epochs. This strategy smoothly transitions the optimization focus from regional pixel coverage to skeletal connectivity.

#### 2.3.3. Candidate Graph Vertex Generation

Morphological thinning is applied to ${\hat{Y}}_{\text{road}}$ to extract a single-pixel-width road skeleton, from which vertices are sampled at a fixed interval of $d_{s}=16$ pixels. Simultaneously, non-maximum suppression (NMS) with a suppression radius of 8 pixels is applied to ${\hat{Y}}_{\text{itsc}}$ to extract local peaks as intersection vertices. These intersections are mandatorily incorporated into the overall vertex set. This yields the final vertex set $V=\{v_{i}\in R^{2}|i=1,\ldots ,N\}$. The selection of these parameters is fundamentally a trade-off: $d_{s}$ balances vertex density against the computational cost of the reasoning stage, while the NMS radius controls how aggressively adjacent intersection responses are merged into a single vertex. These values were held fixed for every experiment reported in Section 3. This procedure effectively suppresses geometric noise and restricts the target space of the subsequent topology reasoning to a set of discrete graph vertices.

### 2.4. Mine-Adaptive Graph Topology Reasoning

**Figure 4:**
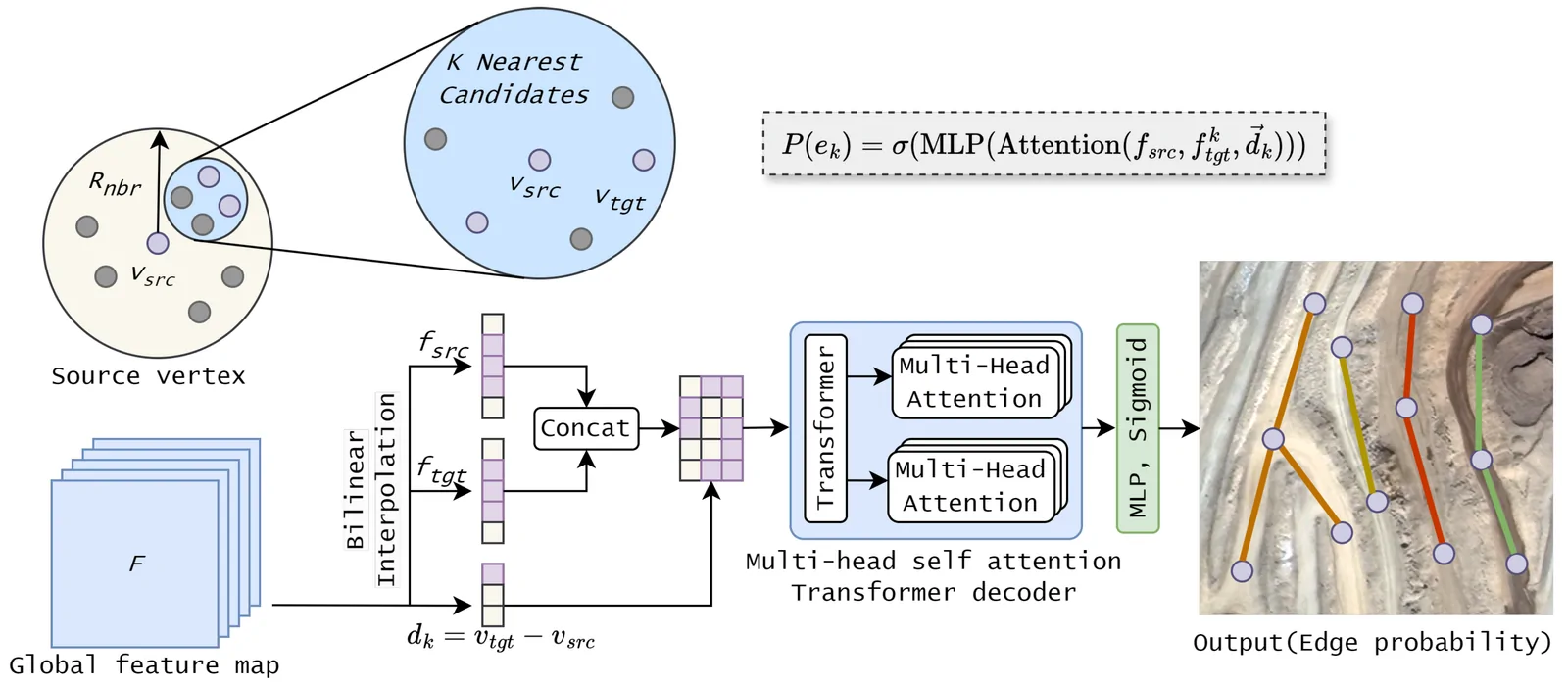
Illustration of the mine-adaptive graph topology reasoning module.

As shown in Figure 4, after extracting the geometric vertices *V*, the topology decoder infers the connectivity relationships among them. For a given source vertex $v_{src}$, a candidate set of K=16 target vertices, denoted as ${\left\{v_{tgt}^{k}\right\}}_{k=1}^{K}$, is searched within a local radius of $R_{nbr}=64$ pixels. The parameter *K* bounds the maximum degree of the reasoning graph, and its value was also held fixed for all experiments. If the number of neighboring vertices exceeds *K*, the candidates are sorted by their Euclidean distance to the source vertex, and only the *K* spatially closest vertices are retained. Conversely, if the count is fewer than *K*, the candidate set is padded with zero vectors. The network employs bi-linear interpolation to sample the source feature $f_{src}$ and target features $f_{tgt}^{k}$ from the global feature map *F*. The relative two-dimensional coordinate offset is computed as ${\vec{d}}_{k}=v_{tgt}^{k}-v_{src}$. The source feature, target features, and positional encodings are subsequently concatenated into a sequence and fed into a multi-head self-attention Transformer decoder. The probability $P(e_{k})$ of an edge $e_{k}$ existing between a pair of vertices is formulated as:

(6) $$P\left(e_{k}\right)=\sigma \left(\text{MLP}\left(\text{Attention}\left(f_{src},f_{tgt}^{k},{\vec{d}}_{k}\right)\right)\right)$$

During the training phase, the ground-truth edge label $y_{k}\in \left\{0,1\right\}$ is constructed according to a strict topological criterion: if the source and target vertices belong to the same connected component in the ground-truth road label, and their shortest centerline path does not traverse a third vertex, it is designated as a positive sample ($y_{k}=1$). Otherwise, it is treated as a negative sample. The topology reasoning process is optimized using a binary cross-entropy loss:

(7) $$L_{\text{topo}}=-\frac{1}{|E|}\sum_{k}\left[y_{k}\text{log}P\left(e_{k}\right)+\left(1-y_{k}\right)\text{log}\left(1-P\left(e_{k}\right)\right)\right]$$

where E represents the set of all candidate edges, and $y_{k}$ denotes the ground-truth edge existence, determined by whether the two vertices are directly connected in the road label.

### 2.5. Domain-Adaptive Differential Fine-Tuning Strategy

A substantial domain gap exists between natural images (e.g., those in ImageNet pre-training datasets) and high-resolution satellite imagery of open-pit mines. As previously shown in Figure 3b, full fine-tuning of a foundation model on limited annotated mining imagery cannot adequately constrain such a massive parameter space, often causing the generic representations that make the model valuable to degrade (catastrophic forgetting). A staged schedule that first stabilizes the randomly initialized decoders and only then admits a strongly reduced learning rate to the deepest encoder blocks is therefore preferable.

Motivated by the need to adapt the Segment Anything Model to the unstructured textures specific to mining environments while preserving its robust generalization capabilities, this study introduces a phased differential fine-tuning strategy:

#### 2.5.1. Frozen Warm-Up Stage

During the initial training phase, all parameters within the SAM image encoder are completely frozen. Only the geometry and topology decoders are updated using a standard learning rate. This process enables the randomly initialized decoding networks to adapt to the basic morphological characteristics of mine roads and achieve preliminary convergence.

#### 2.5.2. Differential Unfreezing Stage

Following this preliminary convergence, the deepest layers of the SAM feature extraction network (e.g., the final two Transformer blocks and normalization layers) are unfrozen. To mitigate catastrophic forgetting, these newly unfrozen layers are assigned a strictly minimized learning rate. Through gentle gradient updates, this strategy adapts the deep receptive fields of the foundation model to the specific geological features of open-pit mines. Consequently, this phased approach effectively alleviates the accuracy bottlenecks frequently encountered when deploying general-purpose visual foundation models in specialized remote sensing domains.

## 3. Experiments

### 3.1. Dataset

Two types of large-scale open-pit mining in China, exhibiting significant topographical and geological variations, are selected as study areas. This selection facilitates the evaluation of the model’s road extraction accuracy and cross-regional generalization capability using high-resolution remote sensing imagery. The primary data source comprises RGB (Red, Green, Blue) composite satellite imagery acquired by the Chinese Jilin-1 (JL-1) constellation on 18 July 2026. These images are geometrically corrected and standard resampled products provided at a spatial resolution of 0.5 m. The first category consists of high-altitude porphyry copper mine clusters in Tibet, specifically encompassing the Yulong, Julong, and Huatailong open-pit mine. With an average elevation exceeding 4000 m, this region exhibits severe topographic relief, resulting in pronounced mountain shadows and illumination variations within the imagery. Furthermore, the unpaved mine roads share highly similar spectral characteristics with the surrounding bare rock and soil, presenting substantial challenges for the continuous extraction of slender road topologies. The second category, serving as a cross-domain test site, is the Nanfen open-pit iron mine in Liaoning Province. This open-pit mine features typical deep-concave multi-tiered bench slopes set against a reddish-brown iron-rich geological background. It exhibits substantial shifts in geomorphological textures, excavation morphologies, and spectral distributions compared to the Tibetan plateau copper mines.

Data partitioning relies on a physical spatial decoupling strategy, effectively mitigating the risks of spatial autocorrelation and information leakage. Large-scale imagery from the Yulong and Julong mines constitutes the primary training and validation sets. The geographically independent Huatailong mine serves as an in-domain, unseen generalization test set which evaluates the model on new, unvisited areas that share the same underlying geological and spectral characteristics as the training data. Conversely, the entire Nanfen iron mine is designated as a cross-domain generalization test set, which tests the model’s adaptability to a completely distinct geomorphological environment and spectral distribution. During the training phase, recognizing that static pre-cropping tends to truncate slender roads and disrupt network topological continuity, the model directly processes large-scale complete images. An online random cropping strategy is employed to dynamically generate training patches of 512×512 pixels. This approach preserves the global topological features of the roads while functioning as a robust data augmentation technique through diverse spatial sampling combinations. The testing phase employs a dual-scale evaluation mechanism. First, 149 standard 512×512 test patches are extracted from the Huatailong test site, facilitating quantitative comparisons with conventional pixel-level segmentation networks under identical local fields of view. Second, the Nanfen test site is retained as a large-scale original image for panoramic inference. This evaluates the model’s macroscopic topological performance in mitigating fragmentation caused by grid stitching, preserving distant intersection connectivity, and generating vectorized road networks for the entire mine.

### 3.2. Implementation Details

#### 3.2.1. ExperimentalSetup and Hardware Configuration

All model training and inference procedures operate within a standardized hardware and software environment, ensuring experimental fairness and reproducibility. Implementation of the algorithm relies on PyTorch (Version 2.1.0)deep learning framework, deployed on a 64-bit Windows workstation (Microsoft, Redmond, WA, USA). The hardware configuration comprises an Intel Core i5 processor (Intel, Santa Clara, CA, USA) and a single NVIDIA RTX 4060 Ti GPU with 16 GB of VRAM (NVIDIA, Santa Clara, CA, USA).

#### 3.2.2. Model Parameters and Training Strategy

The official pre-trained SAM ViT-B variant serves as the visual feature extractor for the backbone network. The training phase employs an online random cropping strategy on large-scale images, fixing the input dimension at 512×512 pixels. The model undergoes 20 training epochs with a batch size of 4 and a base learning rate of 0.001. During the topological connectivity prediction phase, the maximum number of sampled points per block is capped at 512. The encoder parameters participate in the fine-tuning process rather than remaining entirely frozen, thereby adapting to the specific domain characteristics of mine roads.

This specific model configuration was selected as the optimal parameter set following approximately 10 preliminary empirical trials on the validation set, aiming to strike an optimal balance between extraction accuracy and computational efficiency under hardware constraints.

#### 3.2.3. Evaluation Metrics

The stringent requirements for topological correctness in mine road network extraction necessitate the adoption of TOPO F1 and APLS as the core quantitative evaluation metrics. Evaluating local graph structural consistency, the TOPO metric randomly samples seed vertices within the ground-truth road network and compares the structural similarity between the predicted graph and the ground-truth graph across locally reachable subgraphs within a specified search radius. Let $N_{\text{match}}$ denote the number of successfully matched nodes and edges within the local subgraph, with $N_{\text{gt}}$ and $N_{\text{pred}}$ representing the total number of ground-truth and predicted elements, respectively. The TOPO F1 score is formulated as:

(8) $$\text{TOPO}\;F1=\frac{2\times N_{\text{match}}}{N_{\text{pred}}+N_{\text{gt}}}$$

APLS quantifies the disparity between the predicted and ground-truth road networks from the perspective of global routing navigability. Given a ground-truth graph G=(V,E), let L(u,v) denote the shortest path length between two vertices u,v∈V, and $L(\hat{u},\hat{v})$ represent the shortest path length between the corresponding matched vertices in the predicted graph $\hat{G}$. The APLS computation is defined as:

(9) $$\text{APLS}=1-\frac{1}{|V|}\sum_{u,v\in V}\text{min}\left(1,\frac{\left|L\left(u,v\right)-L\left(\hat{u},\hat{v}\right)\right|}{L(u,v)}\right)$$

These two metrics respectively focus on local topological matching accuracy and global path continuity. Consequently, they rigorously reflect topological errors such as structural fragmentations and pseudo-connections in long-distance mine roads, objectively evaluating the practical application value of the model in generating large-scale vectorized road networks.

It is worth noting that TOPO F1 and APLS are continuous comparative metrics rather than absolute threshold-based indicators. Values closer to 1 signify higher structural integrity. Consequently, the satisfactory performance of the proposed method is demonstrated through its substantial relative improvements over baseline models.

### 3.3. Comparison with Existing Methods

To validate the effectiveness of the proposed model, we compare it with several representative road segmentation methods on the self-constructed open-pit mine dataset. Comparison methods include UNet, UNetFormer, OARENet, MSMDFF-Net, Sat2Graph, SegFormer, SAM-Adaptor, and RoadMamba-Net.

#### 3.3.1. Qualitative Comparison

**Figure 5:**
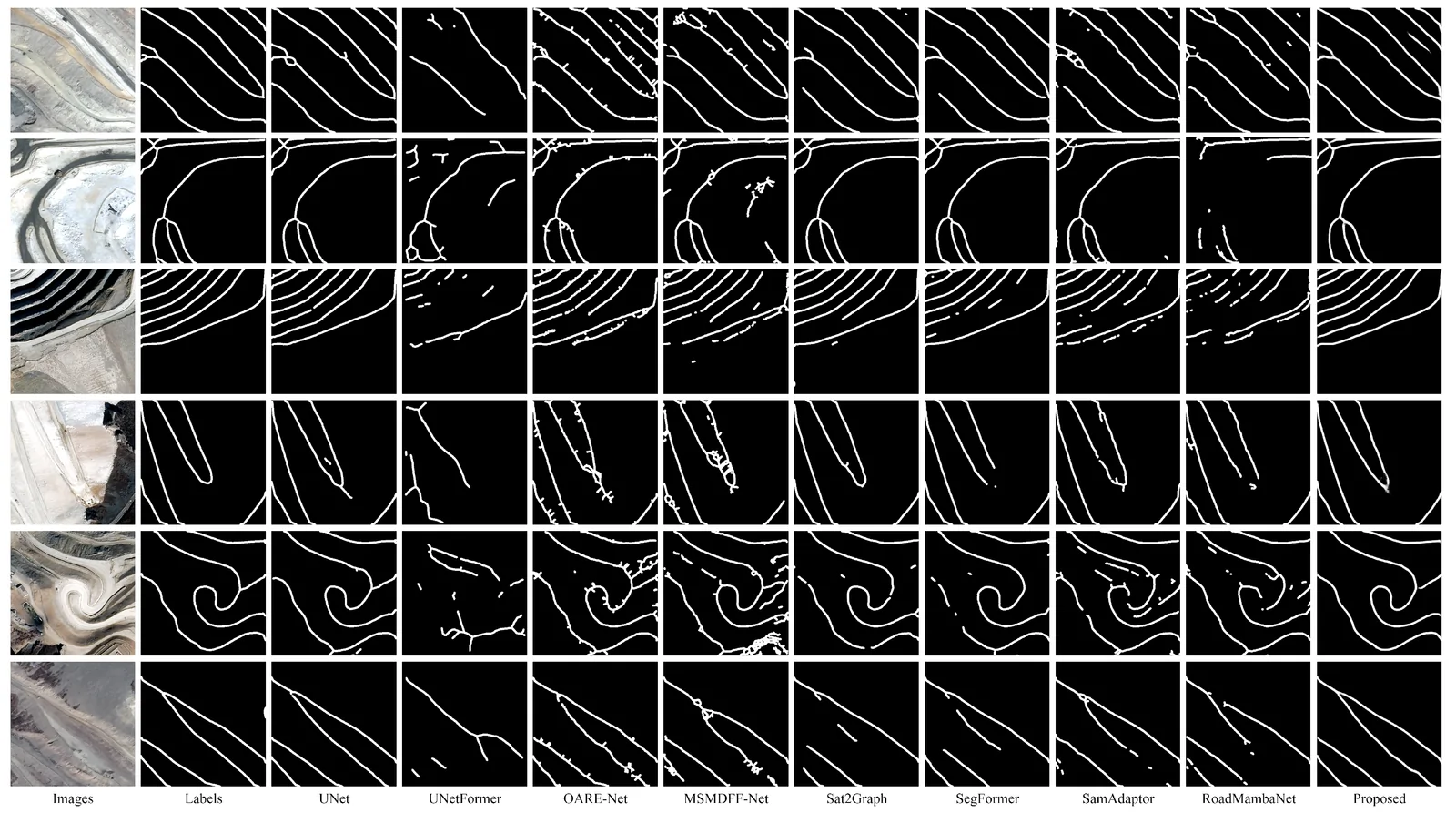
Visual comparison results of the proposed method and baseline models in different challenging scenarios.

A qualitative visual comparison across several typical regions is conducted to evaluate the road extraction performance of different models in complex open-pit mine scenarios. The results are illustrated in Figure 5.

In regions characterized by distinct road features and high radiometric contrast (Figure 5, row 1 and 2), the majority of comparative methods, such as UNet, successfully delineate the main road contours. However, they frequently exhibit topological fragmentations at sharp curves and bifurcations. In contrast, the proposed framework maintains robust road continuity at these critical intersections. This discrepancy primarily stems from the adoption of a graph-based topological reasoning network [48]. Rather than relying on independent pixel-wise classification, the proposed model leverages the Transformer attention mechanism to explicitly infer connectivity edges between nodes, thereby preserving spatial continuity at intersections at the architectural level.

In poorly illuminated mountain shadow regions and overexposed areas caused by intense high-altitude sunlight in Tibet, surface textures undergo severe degradation, diminishing the local boundary gradients between the roads and the surrounding environment. As depicted in Figure 5 (rows 3 and 4), baseline methods such as OARENet and UNetFormer exhibit extensive prediction voids or fragmentations in these regions. This vulnerability arises from their heavy reliance on explicit local edge priors or pixel-level chromatic aberrations. The proposed method, however, preserves the integrity of the road backbone. This resilience is attributed to the illumination robustness acquired by the SAM visual encoder during large-scale pre-training, coupled with the utilization of global contextual semantics, which facilitates the inference of overall road trajectories across localized shadows or overexposed patches.

Under conditions characterized by repeated heavy-duty truck compaction and high spectral similarity between the road surface and the background bare soil (as depicted in Figure 5, rows 5 and 6), the proposed method continues to effectively delineate the road network. Interestingly, UNet also yields commendable visual results in such scenarios. This phenomenon potentially occurs because UNet, trained via end-to-end localized convolutions, develops a certain degree of overfitting to specific dirt road textures. Conversely, UNetFormer exhibits suboptimal performance. In the absence of sufficient pre-training on large-scale annotated mining datasets, its self-attention mechanism struggles to focus on valid local boundaries within highly cluttered mining imagery.

The architectural designs of OARENet and MSMDFF-Net rely heavily on the explicit edge priors typical of urban roads. Consequently, the blurred boundaries and severe width variations of unpaved mine roads render their edge enhancement modules largely ineffective. Furthermore, although Sat2Graph operates as a graph-based model, its reliance on a fixed tensor grid to encode graph structures introduces noticeable quantization errors when processing highly irregular and unstructured mine roads, leading to suboptimal extraction outcomes [49].

Furthermore, while recent architectures such as SegFormer, SAM-Adaptor, and RoadMamba-Net introduce advanced mechanisms, they still exhibit limitations in these complex scenarios. SAM-Adaptor leverages visual foundation priors to achieve relatively robust feature extraction, yet its reliance on dense pixel-level mask prediction limits its global topological navigability. Similarly, RoadMamba-Net and SegFormer struggle to effectively suppress background noise in highly homogeneous reddish-brown environments, frequently producing false-positive fragments.

In summary, by parsing remote sensing imagery into discrete node sets and leveraging attention mechanisms to explicitly infer topological connections, the proposed framework demonstrates that graph-based connectivity modeling consistently outperforms texture-reliant pixel-level segmentation and pure attention-based adaptations in unstructured natural terrains afflicted by extreme illumination and high background noise.

#### 3.3.2. Quantitative Comparison

Quantitative assessments rely on two topology-level metrics, TOPO F1 and APLS, facilitating a rigorous evaluation of model performance. As presented in Table 1, the proposed method achieves the highest scores across both metrics, yielding 90.48% and 45.35%, respectively.

The quantitative results reveal the inherent limitations of conventional methods. For instance, while UNet attains a TOPO F1 score of 88.31%, its APLS score is a mere 36.04%. This phenomenon of high TOPO F1 coupled with low APLS indicates that although convolutional networks successfully identify the majority of road pixels, they struggle to preserve long-range connectivity, where minor structural breaks precipitate a drastic decline in the APLS score. Baseline models such as MSMDFF-Net and OARENet suffer from similar constraints, yielding APLS scores constrained between 34.00% and 40.00%. Recent advanced models exhibit similar topological bottlenecks. For instance, while SAM-Adaptor achieves a commendable TOPO F1 of 80.49% by leveraging SAM’s pre-trained weights, its APLS stagnates at 36.00%. Furthermore, SegFormer and RoadMamba-Net fall behind UNet in TOPO F1, further confirming that standard Transformers or state-space models without explicit topology supervision struggle on this specific task.

The proposed method demonstrates a substantial APLS improvement of 4.6 to 23.9 percentage points over all baseline models. This performance gain is primarily attributed to two architectural designs. First, the integration of the fine-tuned SAM image encoder as the feature extractor provides robust semantic priors. Compared to conventional pre-trained ResNet or CNN backbones, the generalized visual representations acquired from large-scale pre-training exhibit enhanced robustness against the severe spectral homogeneity and illumination variations inherent to mining environments, thereby mitigating background interference at the feature level.

Second, the framework introduces explicit graph modeling and connectivity supervision. Unlike Sat2Graph, which relies on a fixed tensor grid, the proposed topology reasoning module directly establishes node connections via nearest-neighbor search and Transformer attention mechanisms. This formulation enables the network to infer potential edges between vertices across a broader spatial context. Consequently, even when intermediate road segments are obscured or feature gradients degrade, the network leverages sequence attention to span the occluded regions, preserving the structural closure of the road topology. Furthermore, this training paradigm, which explicitly supervises the existence of physical edges, aligns seamlessly with the shortest-path-based evaluation logic of the APLS metric, thereby effectively reducing long-range topological fragmentations.

#### 3.3.3. Statistical Significance Analysis

Statistical validation of the performance improvements over baseline methods relies on a paired significance test conducted on the generalization test set, effectively ruling out the influence of data partitioning biases or random fluctuations. Operating at a significance level of p<0.05, the *p*-values for both the TOPO and APLS metrics were computed, with the results summarized in Table 2. The statistical results indicate that the performance disparities between the proposed method and all baseline models reach a statistically significant level, with all *p*-values remaining strictly below 0.05.

Regarding local topology matching (TOPO), the comparison with UNet, the best-performing baseline, yields a *p*-value of $7.873\times 10^{-3}$ (<0.05). This demonstrates a measurable advancement in predicting road network nodes and local physical connections. Furthermore, comparisons with other baselines, including recent models like SegFormer, SAM-Adaptor, and RoadMamba-Net, result in *p*-values distributed between $10^{-8}$ and $10^{-25}$. These outcomes suggest that the proposed method provides a stable and significant improvement in matching local nodes and edges compared to conventional pixel-level segmentation approaches and generic foundation adaptations.

Concerning global shortest path connectivity (APLS), the *p*-values across all comparative methods consistently fall within the range of $10^{-23}$ to $10^{-25}$. This aligns seamlessly with the preceding qualitative analysis: pixel-level segmentation networks are inherently prone to random fragmentations during the extraction of long-distance continuous road networks. In contrast, by explicitly supervising inter-node connections, the proposed graph topology network demonstrates a more direct architectural alignment with the shortest-path calculation logic underlying the APLS evaluation. Overall, these statistical test results confirm that the observed performance enhancements do not stem from data partitioning variances or random fluctuations. Instead, they are intrinsically attributed to the integrated network design combining the SAM visual encoder with explicit graph structure modeling.

#### 3.3.4. Computational Efficiency Analysis

To comprehensively evaluate the practicality of the models for deployment, we analyze the computational efficiency, including parameter count (Params), floating-point operations per second (FLOPs), and inference speed (FPS). All metrics were measured on a single NVIDIA RTX 4060Ti GPU with a standardized input resolution of 512×512 pixels. The comparative results are summarized in Table 3.

As shown in the table, lightweight convolutional and Transformer models such as UNet (36.88 M, 142.55 FPS) and UNetFormer (2.26 M, 187.78 FPS) exhibit extremely fast inference speeds and low parameter counts. However, as demonstrated in the previous quantitative analysis, this efficiency comes at the severe cost of topological fragmentation (low APLS). Graph-based methods like Sat2Graph incur massive computational overhead (907.61 G FLOPs) due to their complex graph-tensor encoding mechanisms. The proposed RoadTopo-SAM, utilizing the SAM ViT-B backbone, introduces 87.82 M parameters and 113.96 G FLOPs, achieving an inference speed of 25.37 FPS. While heavier than traditional CNNs, its computational footprint is comparable to or more efficient than other foundation-model-based approaches like SAM-Adaptor (114.28 M, 19.67 FPS). Given that open-pit mine road extraction from high-resolution satellite imagery is typically executed as an offline automated mapping task rather than a strict real-time application, the inference speed of 25.37 FPS is highly practical. The substantial improvements in topological navigability fully justify this accuracy-efficiency trade-off.

### 3.4. Ablation Experiment

To rigorously evaluate the core architectural designs of the proposed framework, we conduct ablation experiments on the generalization test set. Specifically, we first validate the necessity of the visual foundation model backbone, and subsequently analyze the contribution of each internal component within the graph topology reasoning module.

#### 3.4.1. Effect of the Visual Foundation Model Backbone

To evaluate the contribution of the Segment Anything Model (SAM) in providing robust semantic priors for this task, we conducted an additional ablation experiment by replacing the SAM visual encoder with a conventional ResNet-50 backbone. The proposed graph generation and topology reasoning modules remained identical to ensure a fair comparison. The experimental results indicate a notable performance decrease when utilizing the conventional CNN backbone: the TOPO F1 decreased to 79.06% and the APLS dropped to 31.55% (compared to 88.31% and 45.35% achieved by the full model with the SAM backbone).

This performance gap reflects the challenges faced by conventional convolutional backbones in unstructured environments. ResNet-50, limited by its localized receptive fields and smaller pre-training scale, exhibits difficulties in distinguishing narrow unpaved dirt roads from the highly homogeneous reddish-brown bare soil and overburden dumps. When optical features are further degraded by mining dust or shadows, the conventional backbone is less effective in providing reliable discriminative cues, leading to noisier and more fragmented node features for the subsequent topology network. Conversely, the generalized visual representations acquired by SAM during its large-scale pre-training help mitigate the impact of these environmental degradations, providing a more stable foundation for topology reasoning.

Regarding computational cost, replacing the SAM backbone with ResNet-50 reduces the inference time from 38.87 ms to 12.30 ms per patch. This observation confirms that the parameterization of the SAM visual encoder constitutes the primary computational bottleneck of the proposed framework. Nevertheless, this computational overhead represents a practical trade-off. While the lighter ResNet-50 architecture accelerates processing speed, it negatively impacts extraction accuracy and topological completeness. In real-world mining navigation, an extracted road network with numerous disconnections (as reflected by the decrease in APLS to 31.55%) poses significant challenges for practical routing applications. Therefore, utilizing SAM as the visual foundation remains a reasonable choice to maintain reliable road connectivity in these complex environments.

**Figure 6:**
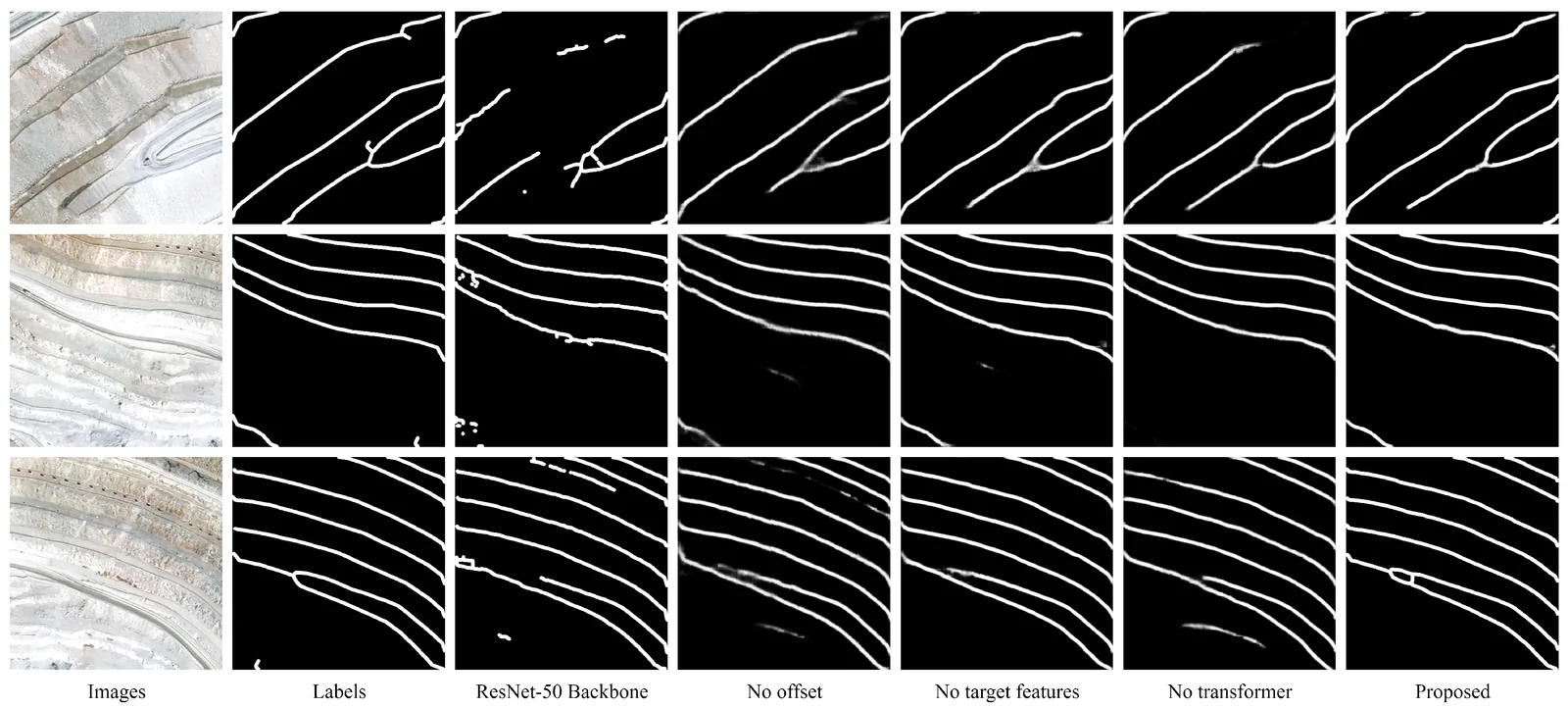
Ablation study on the key components of the proposed method.

Following the backbone validation, we evaluate the specific components of the graph topology reasoning module. Specifically, we remove the target feature aggregation, coordinate offset, and Transformer interaction modules one at a time. The results are summarized in Table 4 and visualized in Figure 6.

#### 3.4.2. Effect of the Coordinate Offset Module

Removing the coordinate offset module decreases the TOPO F1 and APLS scores to 83.40% and 42.19%, respectively. In unstructured open-pit mines, road widths vary significantly and lack distinct geometric centerlines. Relying on fixed grids for feature sampling frequently causes initial graph nodes to deviate from the actual road positions, thereby introducing background noise. The coordinate offset module enables two-dimensional adjustments of sampling coordinates based on local features, correcting the sampling points toward regions with stronger road feature responses. Without this mechanism, feature misalignment becomes pronounced, resulting in a simultaneous decline across both topological metrics.

#### 3.4.3. Effect of the Transformer Module

Excluding the Transformer module yields an APLS of 39.70%, marking the lowest performance among all ablation variants. Road topology reasoning inherently requires evaluating long-range relationships between nodes; relying solely on localized multi-layer perceptrons (MLPs) is inadequate for modeling such complex contextual dependencies. The self-attention mechanism of the Transformer facilitates information exchange among nodes. Consequently, when local features suffer from occlusion or interference, the network can still infer potential connectivity by leveraging semantic information from distant nodes. Because APLS is highly sensitive to long-range path fragmentations, the absence of this module exerts the most prominent negative impact on global routing navigability.

#### 3.4.4. Effect of Target Feature Aggregation

The removal of target feature aggregation reduces the TOPO F1 and APLS to 87.81% and 45.23%, respectively. Robust edge prediction necessitates the simultaneous evaluation of features from both the source and target nodes. When supplied strictly with source node features, the model is prone to generating erroneous connections at intersections or dense operational zones due to the lack of visual confirmation at the target end. Target feature aggregation supplies bidirectional information for connectivity prediction, thereby effectively suppressing a proportion of pseudo-connections.

#### 3.4.5. Computational Cost and Efficiency Trade-Offs

Furthermore, we evaluated the impact of removing these three components on computational cost, measured by the forward inference time per patch (in milliseconds) on a single GPU. The empirical results demonstrate that the proposed modules are inherently lightweight. The baseline full model operates at approximately 38.87 ms per patch. Removing the target feature aggregation and coordinate offset modules yields negligible changes in processing speed, recording 39.00 ms and 38.86 ms, respectively. This indicates that these specific components substantially enhance topological continuity without introducing computational bottlenecks. Conversely, removing the Transformer module provides a measurable reduction in computational overhead, lowering the inference time to 35.95 ms.

Therefore, in resource-constrained edge deployments where processing time is the strictest priority, the Transformer module can be optionally removed to lessen the computational cost. Under relatively benign and well-illuminated mining conditions, this ablated configuration still produces satisfactory extraction results, albeit at the expense of advanced long-range topological reasoning (as reflected by the significant drop in APLS) in heavily occluded areas. Nevertheless, given that the total computational overhead introduced by the complete proposed framework is remarkably marginal (approximately 3 ms), the full model retains highly competitive efficiency for practical applications.

The proposed framework improves spatial alignment via coordinate offsets, provides bidirectional endpoint information through target feature aggregation, and executes global topology reasoning utilizing the Transformer. These three modules explicitly target node localization, relationship verification, and context modeling, acting synergistically to elevate both TOPO F1 and APLS. The ablation results confirm that the removal of any single component induces measurable performance degradation, validating the efficacy of this composite architecture for vectorizing road graphs in complex mining environments.

#### 3.4.6. Effect of clDice Constraint and Intersection Prediction

To comprehensively evaluate the geometry decoding branch, we conducted an additional ablation study focusing on the clDice constraint and explicit intersection prediction. The quantitative results of these specific variants are presented in Table 5.

The experimental outcomes highlight the distinct roles of these two components. When the clDice topological loss is removed (relying solely on standard area-based loss functions), the TOPO F1 score decreases to 77.53%, and the APLS score experiences a substantial drop to 30.94%. This indicates that without explicit skeleton supervision, the network is prone to generating broken predictions along narrow and occluded haul roads, severely damaging long-range routing connectivity.

Conversely, removing explicit intersection prediction primarily affects the local topological accuracy at complex junctions. Without the network explicitly identifying and refining node placements at bifurcations and crossings, the initial graph generation relies on suboptimal vertex candidates. This deficiency leads to a noticeable decline in the TOPO F1 score (dropping to 84.35%), as the model exhibits difficulties in reconstructing valid local physical edges at critical mine intersections. Together, these two constraints ensure that the generated road graph is both locally accurate at junctions and globally continuous along lengthy corridors.

### 3.5. Cross-Domain Panoramic Generalization Testing and Limitation Analysis

Panoramic inference is conducted on large-scale imagery of the Nanfen open-pit iron mine, facilitating the evaluation of the model’s generalization capabilities across distinct domains. Featuring notable discrepancies in geological background, geomorphological morphology, and spectral distribution compared to the Tibetan copper mines, this region provides a rigorous testbed. While the model demonstrates a certain degree of stability in reconstructing the macroscopic road network, it simultaneously reveals specific limitations.

#### 3.5.1. Main Road Extraction and Dynamic Occlusion Handling

**Figure 7:**
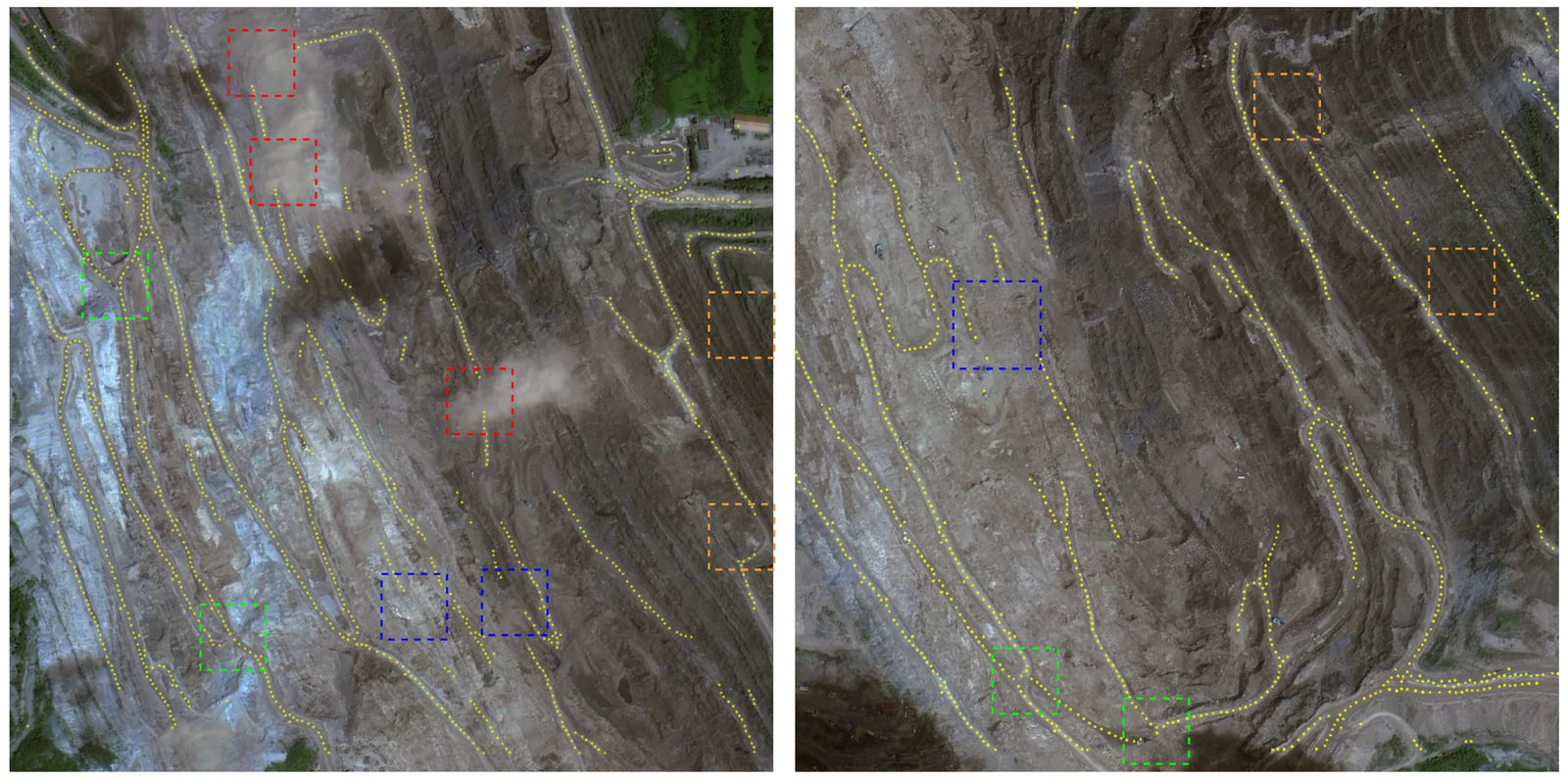
Visualization of region-level cross-domain generalization and limitation analysis on the Nanfen open-pit iron mine. The (**left**) and (**right**) panels present inference results from two distinct, non-overlapping regions within the same mine.

As highlighted by the green bounding boxes in Figure 7, the model successfully recovers the general topology of the main haul roads. The road network maintains its continuity even when localized segments are obscured by large-scale mining vehicles. This resilience is fundamentally linked to the long-range node relationships modeled by the graph structure. While vehicle occlusions typically induce segmentation disruptions in conventional pixel-level methods, the graph model establishes connections spanning across these obscured regions, thereby mitigating the interference of dynamic objects on network continuity.

#### 3.5.2. Spectral Confusion Induced by Geological Discrepancies

Within the dense operational zones of the Nanfen iron mine, the road surface and adjacent gravel piles exhibit highly similar spectral and textural characteristics against the reddish-brown iron-rich background, as indicated by the blue bounding boxes in Figure 7. Due to repeated compaction by heavy-duty machinery, the road boundaries become virtually indistinguishable from the surrounding bare soil relying solely on localized visual cues. The feature distributions learned from the source domain struggle to directly transfer to such highly homogeneous scenarios, yielding incomplete extractions or erroneous connections in localized road segments.

#### 3.5.3. Feature Degradation and Omission of Narrow Roads

Certain extremely narrow roads on multi-tiered bench slopes or long-abandoned dirt tracks remain unrecognized, as marked by the orange bounding boxes in Figure 7. Measuring merely a few pixels in width, these road segments are highly assimilated into the background, yielding weak gradient responses. Under the current input resolution and feature downsampling hierarchy, the model exhibits insufficient responsiveness to such subtle targets. Under zero-shot cross-domain conditions, the sensitivity to scale variations and boundary-blurred targets remains restricted. Future iterations could incorporate higher-resolution shallow branches or frequency-domain enhancement strategies during feature extraction to address these omissions.

#### 3.5.4. Topological Interruptions Under Extreme Occlusion

In regions completely obscured by dense cloud cover, the road network experiences structural disconnections, as shown by the red bounding boxes in Figure 7. The edge reasoning mechanism relies on a predefined neighborhood search radius. When the occlusion span exceeds this radius, the source node fails to identify valid target nodes within its local vicinity. Consequently, the attention mechanism lacks interaction candidates, preventing the establishment of a connection. This phenomenon indicates that the current graph model remains constrained by localized search spaces under extreme physical occlusion. Potential future mitigations for such fragmentations include expanding the search radius, introducing multi-scale graph structures, or integrating external road prior data.

In conclusion, while the model maintains the overall continuity of the macroscopic road network during cross-domain panoramic inference, noticeable deficiencies persist under conditions of spectral confusion, subtle targets, and extreme occlusion. This comprehensive analysis outlines explicit directions for subsequent methodological refinements.

### 3.6. Potential of Image Enhancement Under Adverse Conditions

While RoadTopo-SAM demonstrates robustness relying on the generalized semantic priors of the SAM visual encoder, open-pit mines frequently present challenging imaging conditions. Continuous excavation and heavy-duty truck haulage generate suspended dust clouds, while night-time or twilight operations experience low-light degradation. Under such physical obscuration, directly extracting topological features becomes inherently difficult.

Incorporating image restoration and enhancement techniques presents a potential solution to mitigate these issues. For instance, single-image dehazing utilizing physically motivated priors (e.g., IHDCP) [18] or temporal deep video dehazing could restore the radiometric contrast of dust-occluded road boundaries prior to feature extraction [19]. Furthermore, in poorly illuminated scenarios, fractional-order variational models and hybrid-regularization schemes like VNDHR could assist in recovering structural details in shadowed or night-time environments [21]. Conceptually, these techniques could be integrated into the proposed framework either as a decoupled preprocessing step to standardize the input imagery, or through joint training to make the topology reasoning module more adaptable to environmental degradation. We consider integrating such image restoration modules to be an important direction for our future research, aiming to facilitate all-weather road network monitoring in digital mining applications.

### 3.7. Comparison with Manual Digitization

Manual digitization of a haul road network from high-resolution imagery remains the accuracy ceiling. Human operators can identify individual benches, haul ramps, and maintenance tracks that are physically indistinguishable at the current input resolution, applying prior knowledge of mining operations that a model trained solely on imagery lacks. Consequently, manual digitization is more appropriate when the target area is small, during the initial mapping of a road network where scene interpretation is required concurrently, or when authoritative results with zero tolerance for residual error are demanded.

Its limitations, nevertheless, are structural rather than incidental. Digitization cost grows linearly with the area, making iterative updates for continuously actively mined sites unfeasible under such cost constraints. Furthermore, consistency between operators and between successive campaigns is difficult to control. The proposed method is complementary in exactly these respects: it produces a topologically consistent vector network over an entire mine within a single inference pass. Its output is deterministic for a fixed model, and the residual errors it does make are concentrated in the narrow and heavily occluded segments that are also the slowest for a human operator to trace. Therefore, a rational division of labor in practical operational scenarios involves using the model for bulk extraction and preliminary change detection, reserving manual editing for the small fraction of segments where downstream navigation products demand higher fidelity.

## 4. Conclusions

This study proposes RoadTopo-SAM, a topology-aware vectorized road network extraction framework to mitigate topological fragmentation and domain shift issues in complex open-pit mines. By reformulating road extraction from pixel-level segmentation into a graph generation task, the method leverages the semantic priors of a fine-tuned SAM and explicitly predicts inter-node connections via a Transformer-based topology reasoning module. Experimental results demonstrate that the proposed method achieves state-of-the-art performance, yielding a TOPO F1 of 90.48% and an APLS of 45.35%, with statistically significant improvements over existing CNN and Transformer baselines. Ablation studies verify the roles of coordinate offsets, target feature aggregation, and self-attention mechanisms in maintaining global connectivity. Cross-domain panoramic testing on the Nanfen iron mine indicates the model’s robustness against dynamic vehicle occlusions and geological distribution shifts.

However, certain limitations remain. The model currently processes single-temporal 2D optical imagery, omitting elevation gradients crucial for mining trucks, and relies on a fixed search radius that struggles against extreme large-scale cloud occlusions. To address these limitations, our future work will focus on three main directions. First, we plan to explore multi-modal fusion by integrating Digital Elevation Models (DEM) or LiDAR to extract 3D road topologies. Second, we will develop adaptive, multi-scale graph reasoning mechanisms to dynamically expand the topological search radius and bridge massive occlusions. Finally, to facilitate real-time deployment under severe adverse imaging conditions, we will cascade advanced image restoration front-ends and investigate model lightweighting strategies, such as knowledge distillation. These comprehensive efforts will further propel the practical implementation of autonomous navigation in intelligent mining systems.

## Figures and Tables

**Table 1:** Quantitative evaluation of topology and connectivity on the test set.

Method	TOPO F1 (%)	APLS (%)
UNet	88.31	36.04
UNetFormer	42.91	21.40
OARENet	84.37	40.71
MSMDFF-Net	74.89	34.89
Sat2Graph	81.34	35.07
SegFormer	79.79	34.15
SAM-Adaptor	80.49	36.00
RoadMamba-Net	76.53	28.90
Proposed	90.48	45.35

**Table 2:** Wilcoxon signed-rank test results comparing proposed RoadTopo-SAM with various baselines (p<0.05 indicates statistical significance).

Baseline	TOPO F1		APLS	
	p-Value	Sig. (<0.05)	p-Value	Sig. (<0.05)
UNet	$7.873\times 10^{-3}$	Yes	$1.044\times 10^{-25}$	Yes
UNetFormer	$1.283\times 10^{-25}$	Yes	$1.432\times 10^{-23}$	Yes
OARENet	$6.191\times 10^{-8}$	Yes	$1.525\times 10^{-25}$	Yes
MSMDFF-Net	$3.908\times 10^{-21}$	Yes	$1.044\times 10^{-25}$	Yes
Sat2Graph	$1.288\times 10^{-9}$	Yes	$3.250\times 10^{-25}$	Yes
SegFormer	$3.812\times 10^{-10}$	Yes	$1.011\times 10^{-24}$	Yes
SAM-Adaptor	$3.176\times 10^{-12}$	Yes	$1.525\times 10^{-25}$	Yes
RoadMamba-Net	$3.715\times 10^{-14}$	Yes	$1.525\times 10^{-25}$	Yes

**Table 3:** Computational efficiency comparison of different models (Input size: 512×512).

Method	Params (M)	FLOPs (G)	FPS
UNet	36.88	31.04	142.55
UNetFormer	2.26	11.73	187.78
SegFormer (B1)	15.25	13.68	71.97
MSMDFF-Net	25.81	39.26	73.32
OARENet	9.77	71.44	57.30
RoadMamba-Net	19.83	166.84	160.10
Sat2Graph	55.29	907.61	-
SAM-Adaptor (ViT-B)	114.28	91.40	19.67
Proposed	87.82	113.96	25.37

**Table 4:** Ablation study on the backbone and key components of the proposed framework, including inference time.

Model Variant	Target	Offset	Trans.	TOPO F1 (%)	APLS (%)	Time (ms)
ResNet-50 Backbone	✓	✓	✓	79.06	31.55	12.30
w/o Target	×	✓	✓	87.81	45.23	39.00
w/o Offset	✓	×	✓	83.40	42.19	38.86
w/o Trans.	✓	✓	×	85.48	39.70	35.95
Proposed (SAM Full)	✓	✓	✓	88.31	45.35	38.87

*Note:* ✓ indicates the component is included; × indicates the component is removed.

**Table 5:** Ablation study on the clDice constraint and intersection prediction in the geometry decoding branch.

Model Variant	TOPO F1 (%)	APLS (%)
w/o clDice Constraint	77.53	30.94
w/o Intersection Prediction	84.35	40.61
Proposed	88.31	45.35

## Data Availability

The data presented in this study are available upon request from the corresponding author.
